# The illegal rearing and slaughtering of pigs in the wild on the Mediterranean island of Sardinia favor an increase in the biomass of *Trichinella britovi* in wild boars (*Sus scrofa*) but do not affect the serological prevalence of infection

**DOI:** 10.1186/s13071-023-05927-6

**Published:** 2023-09-11

**Authors:** Ennio Bandino, Maria Angeles Gomez-Morales, Diego Brundu, Manuela Soddu, Alessandra Ludovisi, Piera Angela Cabras, Federica Loi, Antonio Pintore, Edoardo Pozio

**Affiliations:** 1 Istituto Zooprofilattico Sperimentale della Sardegna, Nuoro, Italy; 2 European Union Reference Laboratory for Parasites, Istituto Superiore di Sanità, Rome, Italy; 3 Osservatorio Epidemiologico Veterinario Regionale, Istituto Zooprofilattico Sperimentale della Sardegna, Cagliari, Italy; 4 Laboratorio Fauna Selvatica, Istituto Zooprofilattico Sperimentale della Sardegna, Sassari, Italy; 5 Department of Infectious Diseases, Istituto Superiore di Sanità, Rome, Italy

**Keywords:** *Trichinella britovi*, Wild boar, Free-ranging pig, Epidemiology, Serology, Foodborne parasite

## Abstract

**Background:**

Worms of the nematode genus *Trichinella* are zoonotic pathogens with a worldwide distribution. The first report of *Trichinella* on the Mediterranean island of Sardinia was for *Trichinella britovi*, one of the four species of this genus circulating in Europe, which was identified in 2005 following an outbreak of trichinellosis in humans due to the consumption of pork from pigs reared in the wild. Since then, *T. britovi* larvae have been repeatedly isolated from free-ranging pigs, foxes (*Vulpes vulpes*) and wild boars (*Sus scrofa*) sampled in the central-eastern region of the island (Orgosolo municipality), but have never been isolated from samples from other areas of the island. The aim of this study was to investigate the parasitological and serological prevalence of *T. britovi* infection in wild boars in Sardinia over space [eight wild boar hunting management units (HMUs)] and time (seven wild boar hunting seasons).

**Methods:**

Muscle and serum samples of boars killed in the 2014–2015 to 2020–2121 hunting seasons were collected from eight HMUs of central and south-western Sardinia. *Trichinella* sp. larvae were detected by artificial digestion of predilection muscles. A total of 4111 serum samples of wild boar were collected from the investigated HMUs and tested by enzyme-linked immunosorbent assay as a screening test and by western blot as a confirmatory test using excretory/secretory antigens.

**Results:**

*Trichinella britovi* muscle larvae were detected in six (0.03%) of the 17,786 wild boars tested. All of the *Trichinella* sp.-positive wild boars had been hunted in Orgosolo municipality (central-eastern area of the island), except for one, hunted in a neighboring municipality. An overall serological prevalence of 3.8% (95% confidence interval, 3.3–4.5) was detected by western blot. No statistical differences were detected between the HMUs where *T. britovi* larvae were detected in wild boars, foxes, and free-ranging pigs and those where wild boars, foxes and free-ranging pigs tested negative.

**Conclusions:**

The serological prevalence did not vary between the wild boar populations in which the larval load was detectable by artificial digestion (Orgosolo municipality) and those in which the larval load was below the detection limit. Furthermore, the serological prevalence of anti-*Trichinella* immunoglobulin G in the wild boar populations remained constant during the study period, which covered seven wild boar hunting seasons. As the transmission events (i.e., the serological prevalence) are stable, the high biomass of the parasite in Orgosolo municipality can only have arisen as a consequence of factors independent of its natural cycle, i.e., the presence of a high number of free-ranging pigs, and the concomitant presence of African swine fever, due to illegal pig slaughtering in the field. This epidemiological situation suggests that the natural cycle of *T. britovi* may be influenced by inappropriate pig husbandry and slaughtering practices.

**Graphical Abstract:**

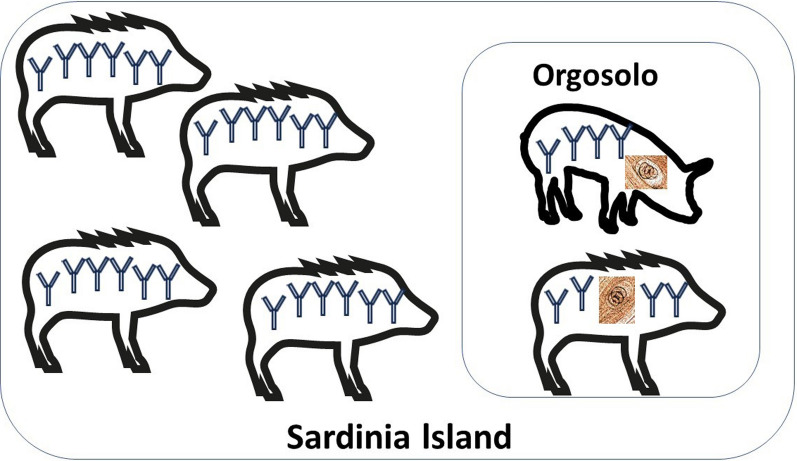

## Background

Foodborne zoonotic nematodes of the genus *Trichinella* are widespread and circulate on most continents. Wild animals play an important role as reservoir hosts of these parasites [1]. Of the 13 described species of *Trichinella*, four (*Trichinella spiralis*, *Trichinella britovi*, *Trichinella pseudospiralis* and *Trichinella papuae*) are often found in naturally infected swine, and *T. spiralis* is the most frequently documented species due to its high reproductive rate and persistence of its larvae in the muscles of infected animals [2–4]. In Europe, although *T. britovi* has mainly been detected in carnivorous mammals, it has also been found in 21.2% of free-ranging pigs and 50% of wild boars (*Sus scrofa*) (International *Trichinella* Reference Center, www.trichinellosis.org). In swine, *T. britovi* shows a low reproductive capacity index and infective larvae survive for no longer than 6 months in muscle [4].

In the Mediterranean islands, *T. britovi* was detected for the first time in a free-ranging pig in Corsica, France in 2004; in the following year, this parasite was detected in Sardinia, Italy during an outbreak of trichinellosis in humans caused by the consumption of sausages made with the pork of a free-ranging pig [5, 6]. Since the discovery of *T. britovi* in these two Mediterranean islands, numerous studies have been carried out to monitor the circulation of this nematode among domestic pigs reared in industrial farms, family farms, or free-ranging in the wild, and in wild boars and red foxes (*Vulpes vulpes*) [6–10]. These studies showed that *T. britovi* was present in free-ranging and backyard pigs, domestic dogs, wild boars, and red foxes, in numerous foci in Corsica, whereas in Sardinia it was only detected in free-ranging pigs, wild boars, and red foxes sampled in the Orgosolo municipality (central-eastern area of the island). In the period 2005–2022, four outbreaks of trichinellosis in humans (a total of 30 cases) and a single case, all due to the consumption of meat from free-ranging pigs or wild boar were reported in Sardinia [7, 9]. Furthermore, an outbreak of trichinellosis in mainland France was also documented, which arose from the consumption of infected pork products imported from Corsica [10]. A subsequent study based on microsatellite analysis indicated that *T. britovi* had probably been introduced into these two Mediterranean islands through two or more independent events that took place during the Middle Pleistocene and Early Holocene [11]. Anti-*Trichinella* antibodies were recently detected in free-ranging black pigs in the Italian island of Sicily, the largest Mediterranean island [12].

The aim of this study was to investigate the parasitological and serological prevalence of *T. britovi* infection in wild boars in Sardinia over time (a total of seven wild boar hunting seasons, from 2014–2015 to 2020–2021) and space (a total of eight wild boar hunting areas in central and south-western Sardinia). Furthermore, the serological prevalence in hunting dogs and the parasitological prevalence of *T. britovi* infection in pigs illegally reared in the wild, and in red foxes, were also investigated to fully evaluate the epidemiology of *T. britovi* in Sardinia.

## Methods

### Study area

**Fig. 1:**
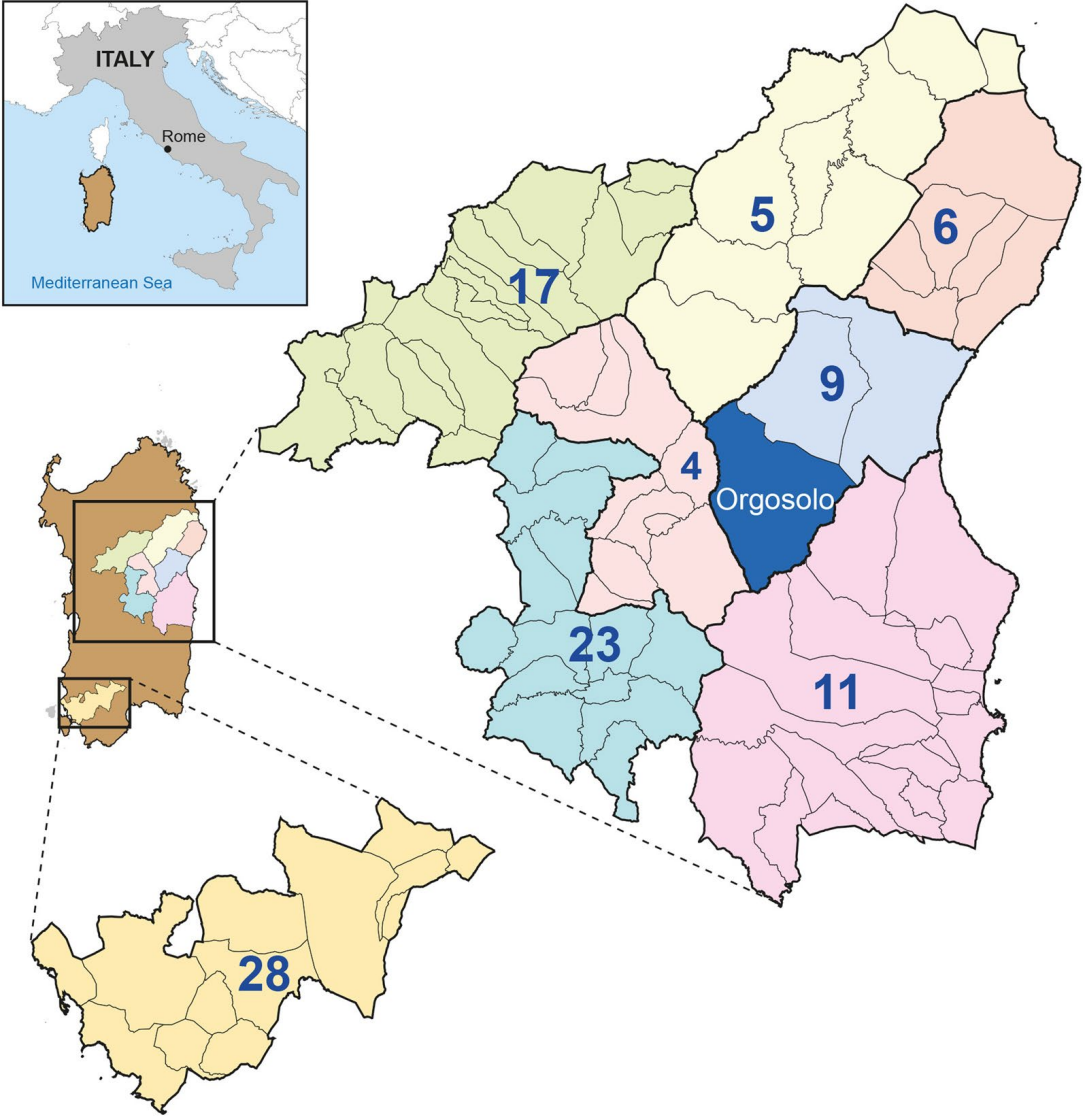
Map of hunting management units in Sardinia where samples were collected from hunted wild animals and from pigs illegally reared in the wild

The central-eastern area of Sardinia, including the Orgosolo municipality, where *T. britovi* has been documented in illegally reared free-ranging pigs as well as in wild animals (i.e., red foxes and wild boar), was chosen as the study area (Fig. 1) [9]. This area is characterized by thick vegetation, mountain ranges and limited water sources, a mild climate during the spring–summer period, and winters characterized by low temperatures and frost. The area is agricultural with a low human population density (17.6 inhabitants/km^2^), and several of the areas inhabited by humans are characterized by social deprivation [13, 14]. African swine fever (ASF) has been endemic in Sardinia since 1978 [15]. Between 2011 and 2016, wild boar health was managed for the control of ASF based on areas in which wild boar are hunted. From 2016 onwards, these areas were revised and named hunting management units (HMUs); the HMUs are bordered by natural barriers that limit the movements of animals [16]. The wild boar hunting season (1 November–31 January) is currently managed within these HMUs to control the spread of ASF, with the obligation that the hunters provide blood and muscle samples from each wild boar hunted [17]. Numerous wild boar HMUs located in the middle of Sardinia can be characterized by the historical presence of pigs illegally reared in the wild [18]. Since 2016, action has been taken against the practice of raising pigs in the wild within the remit of the most recent eradication program for ASF, whereby pigs are culled and then sampled for the presence of ASF and *Trichinella* spp. [19].

### Sampling areas

Seven HMUs located in the central-eastern area of the island, bordering the Orgosolo municipality, were included in this study. The HMUs are located in municipalities of Nuoro province, municipalities of the neighboring Ogliastra province, and some municipalities of Sassari province, which borders Nuoro (Fig. 1). Furthermore, serum and muscle samples collected between 2015 and 2016 from wild boars hunted in a south-western area of Sardinia (Carbonia province), about 130 km from the Orgosolo focus, were used as control samples from a potentially *Trichinella*-free area.

### Detection of* Trichinella* spp. larvae

A total of 17,786 wild boar muscle samples from the seven wild boar hunting seasons from 2014–2015 to 2020–2021 were included in this study. Muscle samples (50 g) were collected from the diaphragm, tongue and/or masseter and tested by artificial digestion at the Istituto Zooprofilattico Sperimentale della Sardegna, in accordance with regulation 2015/1375 of the European Commission [20]. Briefly, 100-g pools of muscle of 20 wild boars (5.0 g per boar) were tested. If the pool of muscles tested positive, 20-g samples from each of five animals were pooled and tested again. When *Trichinella* sp. larvae were detected in a pooled sample from five animals, more 20-g samples from each individual animal were tested again. From 2017 to 2021, a 5- to 10-g sample of tongue or masseter was collected under the ASF eradication program from 1189 pigs illegally reared in the wild in Nuoro province. These pigs were culled by the veterinary services under the protection of the police due to the dangerous social situation [19]. When a pool of 10–20 pig muscle samples tested positive for *Trichinella* sp. larvae it was not possible to trace back the positive pig(s) in the pool due to a lack of additional muscle samples. Twenty grams of the tibialis anterior muscle of 141 red foxes, 5 g of head muscles of eight martens (*Martes martes*), four weasels (*Mustela nivalis*), and a wild cat (*Felis silvestris*) were also tested by digestion. Larvae were kept in 90% ethyl alcohol for further identification to species level by multiplex polymerase chain reaction at the Istituto Superiore di Sanità (ISS), Rome, in accordance with Pozio and La Rosa [21].

As a control, muscle samples from wild boars from south-western Sardinia (Carbonia province) collected between 2015 and 2016 were tested to detect *Trichinella* spp. larvae by digestion in accordance with European Commission regulation 2015/1375 [20].

### Serosurvey of anti-*Trichinella* immunoglobulin G in wild boars and hunting dogs

The sample size needed to detect a minimum serum prevalence of anti-*Trichinella* immunoglobulin G (IgG) of 1% (with a 95% confidence interval; CI) was calculated for each HMU by using EpiTools (copyright 2014 AusVet Animal Health Services) [17, 22, 23]; there was one exception to this, HMU 28, located in south-western Carbonia province, for which the sample size needed to detect a minimum serum prevalence of anti-*Trichinella* IgG of 4% (with a 95% CI) was calculated instead (Table 1). A further 10–20% of samples for every HMU were collected to account for sample dropout. Furthermore, 203 serum samples of hunting dogs from the Orgosolo municipality were included in this study because these act as sentinel animals for the monitoring of infections with *Trichinella* spp. in wildlife [24]. An aliquot of each serum sample was preserved at   -20 °C until use.

**Table 1:** Sample size of wild boar (*Sus scrofa*) serum samples needed to detect a minimum prevalence of 1% with a 95% confidence level of anti-*Trichinella* immunoglobulin G, based on wild boar hunting management unit (*HMU*) surface area and boar density

HMU code	HMU surface (km^2^)	Estimated wild boar population^a^ (no./km^2^)	Required sample size	Number of samples collected
4	471	2799 (5.9)	437	522
5	971	3734 (5.3)	435	657
6	504	2144 (4.2)	421	637
9	615	3438 (5.6)	434	848
11	704	3535 (5.0)	434	391
17	986	5872 (5.9)	447	427
23	734	4360 (5.9)	441	445
28	822	3197 (3.9)	148^b^	184
Total	5807	29,079 (5.2)	3197	4111

^a^The estimated wild boar population per HMU is based on the results of previous studies [17, 22, 23]

^b^The sample size was calculated based on a minimum expected prevalence of 4% with a 95% confidence level

### Serological tests

Wild boar serum samples were first screened by using a commercial enzyme-linked immunosorbent assay (ELISA) kit (PrioCHECK Porcine Trichinella; Applied Biosystem) at the Istituto Zooprofilattico Sperimentale della Sardegna, Nuoro. Then, ELISA-positive sera were forwarded to the ISS in Rome under dry ice for confirmatory testing by western blot (WB) using excretory/secretory antigens, in accordance with Gómez-Morales et al. [25, 26]. Dog serum samples were first tested by an in-house ELISA at ISS, then ELISA-positive sera were tested by using WB with excretory/secretory antigens in accordance with Gómez-Morales et al. [24]. Serum samples were also tested to detect ASF antigen and antibodies [16, 23].

## Results

During the study period (2014–2021), *Trichinella* sp. larvae were detected by artificial digestion in the following: six wild boars (0.03%; 0.6–7.5 larvae/g); six red foxes (4.2%; 6.1–66 larvae/g); six pools of muscles of free-ranging pigs (0.5%; 0.1–43 larvae/g); and one pig slaughtered at home for self-consumption (Table 2). No *Trichinella* sp. larvae were detected in marten, weasel, or wild cat muscles. Most of the *Trichinella* sp.-positive animals originated from the Orgosolo municipality. Only one *Trichinella* sp.-positive wild boar had been hunted in the neighboring municipality of Oliena. All of the larvae were identified as *T. britovi*. As part of the ASF eradication plan, 1189 domestic pigs reared in the wild were killed and tested for *Trichinella* sp. (487 pigs from HMU 9; 483 from HMU 11; 124 from HMU 23; 83 from HMU 6; and 12 from HMU 5). Positive pooled samples were only found for HMU 9.

**Table 2:** *Trichinella britovi* larvae detected in wild animals and in pigs illegally reared in the wild in the seven HMUs from 2014 to 2021

Hunting season	No. positive/no. tested (%)		
	Wild boars	Red foxes	Pigs^a^
2014–2015	2/3081 (0.06)	0/24	1^b^
2015–2016	0/2327	1/35 (2.8)	0
2016–2017	1/2452 (0.04)	1/15 (6.7)	0/26
2017–2018	2/2522 (0.08)	2/31 (6.4)	5^c^/725 (0.7)
2018–2019	0/2795	2/18 (11.1)	0/271
2019–2020	1/3375 (0.03)	0/10	1^c^/125 (0.8)
2020–2021	0/1234	0/8	0/42
Total (%)	6/17786 (0.03)	6/141 (4.2)	6^c^/1189 (0.5)

^a^Free-ranging pigs slaughtered in the field by veterinary services for the eradication of African swine fever

^b^Pig slaughtered at home for self-consumption

^c^Positive pools: the number of *Trichinella* sp.-infected pigs in each positive pool was unknown (for more details, see “Methods”)

An overall serological prevalence of 3.8% (95% CI = 3.2–4.3) was detected by WB (Table 3) for four of the wild boar hunting seasons (2014–2015, 2015–2016, 2016–2017, 2020–2021) from a total of 4111 wild boar serum samples collected for those periods from the investigated HMUs. No statistical difference was found between the serological prevalence in HMU 9, where *T. britovi* larvae were detected in wild boars, foxes, and free-ranging pigs, and the other HMUs (4, 5, 6, 11, 17, 23 and 28), where wild boars, foxes and free-ranging pigs samples tested negative by digestion (Fig. 1; Table 4). Serum samples of 29 (14.3%) hunting dogs tested positive by ELISA, of which three (10.3% of ELISA-positive sera; 1.5% of total sera) were confirmed positive by WB.

**Table 3:** Prevalence of anti-*Trichinella* antibodies detected by enzyme-linked immunosorbent assay (*ELISA positive*) and western blot (*WB positive*) in wild boars hunted in seven HMUs during four of the hunting seasons (2014–2015, 2015–2016, 2016–2017, 2020–2021)

Hunting seasons	Number of tested serum samples (%)	ELISA positive (%, 95% CI)	WB positive (%, 95% CI)
2014–2015	1424 (36)	136 (9.5, 8.1–11.2)	63 (4.4, 3.4–5.6)
2015–2016 and 2016–2017	1608 (39)	182 (11.3, 9.8–12.9)	34 (2.1, 1.4–2.8)
2020–2021	1079 (28)	123 (11.4, 9.5–13.4)	59 (5.4, 4.2–7)
Total	4111	421 (10.7, 9.7–11.7)	156 (3.8, 3.2–4.3)

*CI* Confidence interval; for other abbreviations, see Tables 1 and 2

**Table 4:** Prevalence of anti-*Trichinella* antibodies detected by ELISA and WB in wild boars per HMU during four of the hunting seasons (2014–2015, 2015–2016, 2016–2017, 2020–2021)

Hunting area	No. of serum samples tested (%)	ELISA positive (%; 95% CI)	WB positive (%; 95% CI)
4	522 (13)	61 (11.7; 9.1–14.8)	28 (5.4; 3.6–7.6)
5	657 (16)	54 (8.2; 6.2–10.6)	23 (3.5; 2.2–5.2)
6	637 (15)	74 (11.6; 9.2–14.4)	30 (4.7; 3.2–6.6)
9	848 (20)	105 (12.4; 10.2–14.8)	28 (3.3; 2.2–4.7)
11	391 (10)	54 (14; 10.5–17.6)	15 (3.8; 2.2–6.2)
17	427 (10)	24 (9.7; 6.3–14.1)	11 (2.6; 1.3–4.6)
23	445 (11)	49 (11.0; 8.2–14.3)	18 (4.0; 2.4–6.3)
28	184 (4)	20 (10.8; 6.8–16.3)	3 (1.6; 0.3–4.7)
Total	4111	441 (10.7; 9.8–11.7)	156 (3.8; 3.2–4.3)

For abbreviations, see Tables 1, 2 and 3

## Discussion

The wild boars of the existing population in Sardinia are descended from animals introduced into the island no earlier than the 7th millennium B.C., and probably originate from domesticated pigs that returned to their wild state [27]. Since *T. britovi* had already been present on the island for at least 3000 years [11] when these swine were introduced, it is not surprising that anti-*Trichinella* IgG are uniformly distributed in the present-day wild boar population of Sardinia.

From 2005 to 2021, *Trichinella britovi* larvae were only detected in Sardinia in wild animals (wild boars and red foxes) and in free-ranging pigs in the Orgosolo municipality [6, 7, 9; and data presented here]. The present study shows that the serological prevalence does not vary between the wild boar populations of the HMU in which the larval load is detectable by artificial digestion, i.e., HMU 9, and those of the HMUs in which the larval load is below the limit of detection, 1.0–1.9 larvae/g [28]. These epidemiological data agree with laboratory data that showed that *T. britovi* larvae survive in swine muscles for up to 6 months after infection, whereas the antibody response persists for at least 2 years after infection [4].

The fact that the biomass of *T. britovi* in the Orgosolo municipality (HMU 9) was higher than that of the other hunting areas with similar antibody prevalence, and where the parasite has never been detected in the muscles of pigs reared illegally in the wild or in wild animals, indicates that the pathogen’s transmission events among susceptible animals were constant over time (four hunting seasons) and space (eight HMUs of central and south-western Sardinia). As the transmission events were stable, the presence of a high biomass of the parasite in the Orgosolo municipality can only be due to factors independent of those of the natural cycle, i.e., the presence of a high number of free-ranging pigs and the concomitant presence of ASF due to their illegal slaughtering in the field. ASF has been endemic in Sardinia since 1978, and there were active foci of this virus in the investigated areas until 2018 [13, 15, 29]. In HMU 9, cyclical outbreaks of ASF reported in domestic pigs illegally reared in the wild were associated with high mortality and the presence of infected carcasses in the wild and thus the possibility of more, new *T. britovi* infections in scavenging animals.

From 2005 to 2021, as many as 25 domestic pigs (mainly sows) illegally reared in the forest of the Orgosolo municipality for family self-consumption were found to be positive for *T. britovi* larvae [7, 9; the present study]. The higher larval biomass and prevalence of infection in pigs (0.1–43 larvae/g; 0.5%) than wild boars (0.6–7.5 larvae/g; 0.03%) indicate the impact of free-ranging pigs on the epidemiology of *T. britovi* in the Orgosolo municipality.

The parasitological prevalence detected by artificial digestion (0.03%) and the serological prevalence of anti-*Trichinella* IgG detected by WB (3.8%) in wild boars in Sardinia are similar to the those (0.07% and 2.2%, respectively) reported for wild boars in five continental Italian regions where *T. britovi* is circulating among susceptible wild animals [26]. Serological tests do not allow recent infections to be distinguished from past ones, but we assumed that most of the wild boars found to be seropositive only harbored residual antibodies and had no infective larvae in their muscles.

The fox is one of the main natural reservoirs of *T. britovi* in Europe. *T. britovi* prevalence in this carnivore in the present study (4.2%) is similar to that detected in other Italian regions [30], but much higher than the average prevalence in red foxes of the European Union in recent years [31].

The anti-*Trichinella* IgG prevalence of 1.5% of hunting dogs in Orgosolo municipality confirms the results of previous investigations, which showed that they can act as sentinel animals for the monitoring of *Trichinella* sp. infections in wildlife [24]. A bias of this study was the lack of testing of sera of hunting dogs from the HMUs where *T. britovi* larvae have never been detected in wild boars or foxes.

From 2002 to 2023, a total of 185 human cases of trichinellosis reported in Italy were due to the consumption of wild boar meat from hunting, of which 155 cases were caused by *T. britovi* and 30 by *T. pseudospiralis* [32–34; unpublished data,MA GM]. The presence of *T. britovi* in wild boars represents a threat to hunters and their families because the carcasses of some of the hunted animals escape official controls and thus are considered a health risk.

## Conclusions

The presence of ASF in the population of wild boars in Sardinia and the coexistence of these animals with herds of pigs reared in the wild that have escaped any veterinary control have certainly favored an increase of the *T. britovi* biomass in the last two decades, which has led to outbreaks of trichinellosis among humans. Artificial digestion can be used to determine the larval load of infected meat that is sufficient to cause clinical infection in individuals who eat it [28]. However, this diagnostic method cannot detect low parasitic loads that are of sufficient magnitude to perpetuate the cycle of *T. britovi* in the wild. The results of this investigation suggest that, in an insular context under a Mediterranean climate, *T. britovi* can circulate in susceptible wildlife at a very low prevalence and, notably, the larval load is lower than that which can be detected by artificial digestion, as has also been observed in Sicily. However, inadequate pig rearing and slaughtering practices can modify the natural cycle, as shown in the municipality of Orgosolo. The results of this study indicate the following: that programs need to be implemented throughout the region for the surveillance of *T. britovi* in animals that are hunted and in farmed pigs; that the breeding of pigs in the wild needs to be prohibited; and above all, educational programs need to be implemented for breeders, hunters, and consumers of boar meat and pork. Fox carcasses left in the field after the animals have been skinned, and the discarding of entrails/offal and other parts of hunted wild boars in hunting areas, and those of pigs following their illegal slaughter, favor the transmission of the parasite among scavenging omnivorous and carnivorous mammals. It was shown that the education of hunters and the organization of a collection and storage service for slaughter leftovers led to a drastic reduction in the prevalence of *T. spiralis* infection in an animal population [35]. Thus, health authorities should provide a service for the disposal of animal carcasses and offal, and for the training of hunters and pig owners for the management of waste and byproducts, in accordance with the relevant European Union regulations.

## Data Availability

Data supporting the conclusions of this article are included within the article and its additional files. The raw data can be obtained from the ISS (MAGM; mariaangeles.gomezmorales@iss.it) and the Istituto Zooprofilattico Sperimentale of Sardinia, Nuoro (EB; ennio.bandino@izs-sardegna.it).

## Abbreviations

ASF: African swine fever
CI: Confidence interval
ELISA: Enzyme-linked immunosorbent assay
HMU: Hunting management unit
ISS: Istituto Superiore di Sanità
WB: Western blot
